# Secondhand Smoke Exposure and Brain Health Indicators in Cuban Preschoolers

**DOI:** 10.3390/toxics13010062

**Published:** 2025-01-17

**Authors:** Yaser Ramírez Benítez, Miriela Díaz Bringas, Rodneys Mauricio Jiménez-Morales, Ijang Bih Ngyah-Etchutambe, Linda S. Pagani

**Affiliations:** 1 Centro Universitario Municipal Rodas, Universidad de Cienfuegos, Cienfuegos 59430, Cuba; 2 Centro de Rehabilitación del Neurodesarrollo de Cárdenas, Matanzas 42110, Cuba; mirieladiaz71@gmail.com; 3 Instituto Superior Antonio Ruiz de Montoya, Misiiones 3300, Argentina; mauricio770927@gmail.com; 4 Department of Educational Psychology, University of Buea, South West Region, Buea P.O. Box 63, Cameroon; ijangetchu@gmail.com; 5 École de Psychoéducation, Université de Montréal, Montréal, QC H3C 3J7, Canada; linda.s.pagani@umontreal.ca

**Keywords:** secondhand/household smoke, environmental smoke, tobacco, nicotine, family environment, child neurotoxicity, brain health

## Abstract

Secondhand smoke affects nearly 40% of children worldwide, leading to serious health and behavioral problems. Being neurotoxic, it poses potential risks for child health and learning. In Cuba, there is limited research on the association of secondhand smoke with children’s brain health, especially in vulnerable populations like young children at home. The overall purpose of this study is two-fold. First, we determined the relationship between household smoke exposure and risks to brain health in Cuban children. Second, we analyzed the role of family environment factors, such as socio-economic status, in our estimates. Although this research represents the first investigation of its kind in Cuba, we expect to find evidence of neurotoxic associations with household smoke. We collected data between 2015 and 2018 using the medical records of 627 Cuban preschool children to explore the link between brain health indicators and exposure to tobacco smoke at home. We assessed archival reports on parental smoking, duration and frequency of exposure, and several indicators of brain health, including executive function, language development, sleep quality, and fluid intelligence. The findings indicate that exposure to tobacco smoke at home has a negative association with children’s brain health, affecting both the cognitive (executive and linguistic functions) and non-cognitive aspects (sleep quality) of child development. Continuous exposure (five to seven times per week) and transient exposure (two to three times per week) were found to be more negatively related to sleep quality than in cognitive functions, particularly in children of middle socio-economic status. This highlights the need to implement parental information campaigns in Cuba.

## 1. Secondhand Smoke Exposure and Brain Health Indicators in Cuban Preschoolers

The World Health Organization estimates that 40% of children worldwide are exposed to secondhand tobacco smoke, either at home or in public places [1]. This exposure can lead to a range of health problems in children, including acute respiratory infections, asthma, middle ear infections, and sudden infant death syndrome [2]. Thus, it is crucial to adopt a comprehensive approach to promote brain health in children growing up in vulnerable communities.

As defined in Jenssen et al. [3], sources of tobacco smoke (and vapor) include e-cigarettes, cigarettes, cigars, smokeless tobacco, hookahs, pipe tobacco, heated tobacco products, and nicotine “tobacco-free” pouches. Environmental tobacco smoke refers to contextual tobacco smoke inhaled by both the active firsthand smoker and others nearby [4]. It consists of 85% sidestream emissions discharged from a burning cigarette and 15% mainstream emissions discharged from the smoker(s). Sidestream smoke is considered more toxic than its mainstream counterpart, given that it comprises a comparatively greater concentration of dispersed respirable pollutants over a longer exposure period [5]. There is a consensus that it comprises at least 250 toxic chemical gases, including carbon monoxide, hydrogen cyanide, butane, ammonia, benzene, and toluene [1]. Important toxic metal ingredients include lead, chromium, arsenic, and cadmium. These substances, inhaled by both active smokers and their entourage, are associated with morbidity and mortality [4].

Secondhand smoke refers to fumes (and vapor) emitted from a tobacco product or exhaled metabolites from smokers that are inhaled by a person exposed to the same environment [5]. People are then exposed to such toxins by direct contact and dermal absorption, ingestion, and/or off-gassing and inhalation [1]. Secondhand smoke becomes thirdhand smoke, which tends to interact with other substances in the environment, thus yielding more chemical-based and metal-based pollutants [1]. Thirdhand Smoke occurs when such toxic gases take a solid form and then fall or are absorbed onto surfaces [1]. There is no established safe level of environmental exposure [4].

For children, secondhand (and vapors) and thirdhand smoke occur in domestic spaces where families live and breathe [2]. The home represents an important risk context for some children exposed to household smoke. Infants and young children are specifically vulnerable, with insidious exposure at home due to smoking parents or caregivers [1]. In fact, given their underdeveloped neurological, immune, and respiro-circulatory systems, children account for the largest global disease burden associated with prenatal and postnatal household smoke [5].

There is an increasingly robust association between tobacco smoke exposure before and after birth and executive function problems in children, adding to current and future disease burden estimates in public health [6]. It has been established that environmental smoke exposure is harmful to child development [6,7]. The associated risks include emotional and behavioral problems [8,9,10]. It is negatively associated with executive function development and fluid intelligence [2,11]. These associations present risk factors for less academic and personal success in human development [2].

The World Health Organization defines brain health as an optimal cortical state in which humans can reach their full potential throughout their life course [12]. In practical terms, brain health includes aspects such as concentration, memory, stress management, emotional regulation, decision-making, and sleep quality [13]. Promoting cortical well-being involves adopting healthy exposures and lifestyle habits, such as a balanced diet, regular exercise, adequate rest, stress management, and activities that stimulate the mind to promote optimal synaptic function and prevent mental health problems. Despite the clarity of the concept, pediatric and family medical care do not often intervene in the unhealthy aspects of the environment with parents [13]. Information campaigns start in the doctor’s office by teaching parents that secondhand and thirdhand smoke in houses, cars, and other enclosed spaces can negatively affect children’s behavior and brain health [3].

From a multidisciplinary perspective, brain health is conceptually related to several aspects of child achievement and mental health, which refer to an interdisciplinary interplay of innate, psychological, social, and ecological factors in development [13,14]. Brain health is represented by indicators of both protection and vulnerability [15], by encompassing predictors of health and well-being [16]. Sleep plays a critical role in brain health by facilitating the elimination of metabolic waste and promoting optimal brain development. Sleeping between 10 and 14 h from infancy to kindergarten has been associated with good health in later childhood [1]. Sleep has a significant relationship with 88 health indicators [17]. It has been observed that both adult and adolescent smokers often have difficulty falling asleep, experience less quality sleep, and tend to sleep fewer hours during the night compared to nonsmokers [18]. Similarly, it has been observed that passive smokers also experience difficulties in falling asleep, such as frequent snoring during the night, sleep apnea, and sleep restlessness, including in adolescence [19]. This especially applies to children under the age of 7 years [20].

From a holistic perspective, executive functions are essential for children’s social and emotional development. They enable effective self-regulation and are predictive of academic success and mental health [21]. Executive functions are more important for school readiness than the intelligence quotient and are predictive of academic competencies throughout the school-age years [22]. Children and adolescents exposed to household smoke represent a vulnerable population, especially in low- and middle-income countries. Therefore, given its critical role in academic success and mental health [23,24], it would be imperative to prioritize executive function to improve child school readiness and academic performance in such contexts.

There are economic, political, and social reasons to believe that Cuban children represent a vulnerable population despite its consolidated education and health system context compared with other countries in Latin America [25,26,27,28]. A 2010–2011 study in Cienfuegos City (Cuba) revealed that 30% of men and 20% of women smoke [29]. According to the Tobacco Atlas estimates, approximately 18.5% of parents smoke in Cuba [30]. According to the World Health Organization, approximately 18,000 people die each year in Cuba from tobacco-related diseases, accounting for about 20% of all deaths in the country [1]. In addition, tobacco is estimated to be responsible for about 10% of all deaths from cardiovascular disease and 20% of all cancer deaths in Cuba. The Global Youth Smoking Survey by the Pan American Health Organization indicates that approximately 11.5% of adolescents aged 13–15 use tobacco, with 8.7% smoking cigarettes [31]. It is estimated that for every adult smoker in Cuba, one to two children are exposed to secondhand tobacco smoke [31]. Disconcertingly, over 55% of children under 15 are subjected to tobacco smoke, especially in urban settings [31]. These figures illustrate the significant impact of tobacco on health and mortality in the country [32,33,34].

The estimates of child exposure increase exponentially when we add the effect of assortative mating practices, where two people with common characteristics tend to form couples [35]. If one parent smokes, it is likely that the other smokes. To combat household smoke, Cuba’s public health system has teamed up with the education sector to implement campaigns targeting tobacco prevention among adolescents, pregnant women, and the community. It also has a preschool program where children attend in smoke-free environments, away from home [36]. These initiatives aim to lower smoking-related illnesses and protect children and teenagers from household smoke [31,37,38,39]. As such, we would expect that smoking parents in Cuba will be more aware of how harmful household smoke is to the holistic development of the child. However, research on the effects of household smoke on early childhood brain health in Cuba is limited, highlighting the need for studies on how maternal smoking influences children’s brain health and behavior.

The overall purpose of this study is two-fold. First, we aim to examine the relationship between household smoke exposure and risks to brain health in Cuban children. Second, we aim to examine the role of family environment factors, such as socio-economic status, in this equation. Few studies have addressed children in LMICs. None have examined indicators of financial hardship within such risk associations. Although this research represents the first investigation of its kind in Cuba, we expect to find evidence of neurotoxic associations with household smoke.

## 2. Methods

### 2.1. Participants

The study participants were sourced from medical records of children in the “Family and Community Based Early Neurodevelopmental Care” project, managed by the Neurodevelopmental Rehabilitation Center of Cardenas “Rosa Luxemburgo” at the University of Matanzas’ Faculty of Medicine in Cuba. The study analyzed a total of 1000 clinical records corresponding to the period from 2015 to 2018 in the center. The inclusion criteria were as follows: (1) children evaluated with the Luria Inicial Neuropsychological Battery between 2015 and 2018, and (2) a medical interview for the discovery of the participant’s full medical history (family demographics, substance use and smoking habits, sleep, diet, behavior, development, and education). Exclusion criteria included (1) children with severe intellectual disability and (2) a diagnosis of autism or clinical impression of this syndrome. At the end of the study, 627 medical records of 6-year-olds were selected, of which 263 were girls, representing 42%, as detailed in Table 1.

### 2.2. Ethical Approval and Informed Consent

The Scientific Council of the Neurodevelopmental Rehabilitation Center of Cardenas “Rosa Luxemburgo” at the University of Matanzas’ Faculty of Medicine in Cuba approved the research and authorized the review of the clinical histories of the period analyzed, according to the guidelines of the Declaration of Helsinki.

### 2.3. Measurements: Predictors

*Household smoke exposure (SSE)*: We generated a composite variable related to household smoke exposure by assessing the mother’s smoking habit, as well as the time and frequency of child exposure throughout the week. During a medical interview, mothers answered a series of questions: (1) maternal smoking, “Do you smoke at home?” (0 = absent, 1 = present); (2) child exposure to maternal smoke, “Is your child near you when you smoke?” (0 = absent, 1 = present); (3) days of exposure to household maternal smoke, “How many days per week do you smoke at home?” (0 = no exposure, 1 = exposure of 2 to 3 days per week, 2 = exposure of 5 or more days per week); (4) hours of exposure to household maternal smoke, “How many hours per day do you smoke at home?” (0 = low exposure, ≤1 h per day; 1 = medium exposure, between 2 and 3 h per day; 2 = high exposure, 4 h or more per day). Based on these answers, we created an index of household exposure risk. The total score on the index was obtained by adding all the values collected (answering yes on questions 1 and 2 and scores on questions 3 and 4). Finally, the values of the four questions were added, and the total was divided by 4. This allowed the classification of children into three categories: never exposed, transient exposure (2 to 3 h per day and 2 or 3 days per week), and continuous exposure (more than 4 h per day and 5 or more days per week). This classification has been used in other studies [40,41].

### 2.4. Measurements: Outcomes

*Brain Health:* Four key indicators of children’s brain health were identified: executive function, oral language development, sleep quality, and fluid intelligence. These indicators were used to create a composite variable. The total value of the variable to be used in the equation was obtained by adding all the values collected from the four factors and dividing the result by 4.

*Executive function and oral language development:* These were assessed using the Initial Luria Battery (BLI), a neuropsychological test adapted for Cuban preschool children [42]. Internal consistency was adequate for 14 subtests (α = 0.86) and excellent for 10 variables (α = 0.92), according to Ramírez et al. [43]. Data from five executive function subtests and two immediate memory subtests of the BLI [42] were used to define three variables: working memory, inhibition, and cognitive flexibility, based on the theoretical criteria of Diamond [44]. The equations used to calculate these variables were as follows:

- Working memory (WM) = 1/4*verbal memory scores + 1/8*visual memory scores + 1/8*manual motor scores + 1/8*right-left orientation scores + 1/8* gesture and praxis scores + 1/8*verbal regulation scores + 1/8*spatial orientation scores.
- Inhibition (HI) = 1/4*verbal regulation scores + 1/8*manual motor scores + 1/8*right-left orientation scores + 1/8*gesture and praxis scores + 1/8*spatial orientation scores + 1/8*verbal memory scores + 1/8*visual memory scores.
- Cognitive flexibility (CF) = 1/4*spatial orientation scores + 1/8*verbal regulation scores + 1/8*manual motor scores + 1/8*right-left orientation scores + 1/8* gesture and praxis scores + 1/8*verbal memory scores + 1/8*visual memory scores.

The equations were developed to capture the variability of the seven subtests in three different variables, assigning different weights to each according to its characteristics. Greater weight was given to working memory with the verbal memory subtest, inhibition with the verbal regulation subtest, and cognitive flexibility with the spatial orientation subtest. This analysis was carried out using SPSS 25 software, and the weights were not arbitrary but based on theoretical descriptions of the BLI and previous experience in the Cuban context [43].

*Oral language development:* The results of five language function subtests of the BLI [42], including object and figure naming, phonemic listening, picture vocabulary, similarities–differences, and oral numerical operations, were integrated into a single variable representing language development. This integration was carried out according to the theoretical criteria proposed by Narbona and Chevrie-Muller [45], which include linguistic, phonological, morpho-syntactic, semantic, and pragmatic components. To calculate this variable, all the data were summed and divided by the number of subtests.

*Fluid intelligence:* An adapted version of the Raven’s Progressive Matrices test was used to assess fluid intelligence in preschoolers, which has an internal consistency of (α = 0.89) [46]. This adapted test consists of 36 non-verbal problems that require the completion of incomplete figures, with one point scored for each correct answer, according to Cuban standards. Validation of this version is found in Ramírez et al. [46].

*Sleep quality:* This was assessed by a medical interview with the mother, which included two questions: (1) hours of sleep per night (How many hours does your child sleep?) (0 = insufficient (≤6 h), 1 = sufficient (≥7 h) and (2) restlessness during sleep (Does your child often move during sleep?) (0 = absent, 1 = present). The classification of sleep quality follows the guidelines set by the American Academy of Sleep Medicine, which recommends that children aged between 6 and 12 years ideally sleep between 9 and 12 h per night for optimal health and cognitive functioning [47]. Buxton and Marcelli further suggest that 7 to 8 h of sleep is associated with the greatest health benefits and optimal cognitive performance [48].

### 2.5. Measurements: Confound Controls

*Family factors*: Parents completed a questionnaire that included the following indicators: (1) the salary of each family member eligible to work, (2) the number of family members in the household, and (3) maternal education. The socio-economic status of the family is defined as the sum of all salaries divided by the number of family members. The average monthly salary values allowed the family to be classified as low (CUP ≤200), medium (CUP 220–399), and high (CUP > 400), according to the category “Average monthly salary in Cuban government entities” of the 2018 Statistical Yearbook of Matanzas. In analyzing the country’s economic conditions on the *ECONOMYi* variable, we considered both the influence of specific variables (family socio-economic status) and general indicators of the Cuban economy (GDP per capita_2022_ = 56.49, Unemployment Rate_2022_ = 1.4, GINI Index_2022_ = 0.40, Human Development Index_2019_ = 0.78, and Inflation Rate_2023_ = 45%). These indicators were used because they accurately reflect the real conditions of our social environment. Maternal education was coded as follows: 1 = a high level of education, e.g., university, and 0 = a medium level of education, e.g., high school.

*Child Factors*: During a medical interview, the mother answered a series of questions about the child’s behavior: (1) plays with other children (0 = recreational games (free play activities); 1 = adult-directed games (games based on specific development objectives), (2) child discipline (0 = bad and 1 = good), and (3) daytime restlessness (0 = absent and 1 = present). The activity theory model highlights that free play and adult-directed play are crucial for child development, enhancing physical, cognitive, and socio-emotional growth. However, adult-directed play is more likely to meet age-appropriate developmental goals [49].

### 2.6. Data Analytic Strategy

Our data analytic procedures implement ordinary least squares regression to estimate indicators of executive function from maternal household smoke. We first determined the relationship between household smoke exposure and risks to brain health in Cuban children and then analyzed the role of socio-demographic factors. Few studies have addressed children in LMICs.

First, using transformed z-score data, we conducted a series of ordinary least squares regressions to test the proposed linear relationship between household smoke, brain health, and other confounding factors such as individual child and family characteristics.

Then, specifically, for each individual case (individual_i_), we regressed the composite brain health variable score (*BRAINi*) on household smoke exposure (*SMOKEi*). In this first regression, the first objective was answered: we determined the relationship between brain health and household smoke in children with transient and continuous exposure.

Subsequently, to ensure unbiased estimates, our fully controlled model accounted for potential omitted variable bias and confounding factors arising from the country’s economic conditions over the past five years (*ECONOMYi*), pre-existing and concurrent family factors (*FAMILYi*), and individual child characteristics (*CHILDi*) that could have a significant influence on our key variables. This last analysis allowed us to analyze the influence of family and economy in a completely controlled equation.

BRAINiAge = a1 + ß1 SMOKEi + γ1 ECONOMYi + γ2 FAMILYi + CHILDi + eit,

where a1 and eit represent the intercept and stochastic error, respectively:

*BRAINiAge* = 0.3*executive functions + 0.3*language development + 0.15*fluid intelligence + 0.15*sleep + 0.10*age.

*SMOKEi* = mother smoking + child exposed to household smoke + number of days exposed to household smoke in the home + number of hours exposed to household smoke in the home.

*ECONOMYi* = 0.2*Family socio-economic status + 0.3*GDP per capita_2022_ + 0.1*Unemployment rate_2022_ + 0.1*GINI index_2022_ + 0.2*Human Development Index_2019_ + 0.1*Inflation rate_2023_.

*FAMILYi* = socio-economic status of the family + mother’s education + number of members.

*CHILDi* = fluid intelligence + behavior + gender + age.

## 3. Results

Table 1 reports descriptive statistics from a sample categorized into three groups based on exposure to household smoke at home: never exposed (n = 535), transiently exposed (n = 25), and continuously exposed (n = 67). The average age of children in the never-exposed group was 72 months (SD = 8.36), while the transient and continuous exposure groups had mean ages of 82.50 months (SD = 4.57) and 80.82 months (SD = 4.96), respectively.

In terms of sex distribution, 58% of the never-exposed children were boys, compared to 68% in the transient exposure group and only 36% in the continuously exposed group. Maternal smoking rates varied significantly: 100% of mothers in the continuous and transient exposure group smoked, while the rest did not smoke (the never-exposed children group).

Regarding exposure duration, 10% of the continuously exposed children experienced high levels of exposure (≥4 h), compared to 8% in the transient group. Both groups had children exposed for 2 to 3 h weekly, with 24% in the transient group and 25% in the continuous group. The household exposed risk index indicated that 100% of the children with continuous exposure had higher values (≥1), while only 32% of the children with transient exposure had similar values.

On brain health assessments, the never-exposed group had a mean score of 4.34 (SD = 2.55), while the transient and continuous groups scored 4.80 (SD = 1.63) and 5.78 (SD = 2.39), respectively. Working memory scores were notably higher in the continuously exposed group, averaging 14.89 (SD = 2.21), compared to 12.87 (SD = 2.62) in the never-exposed group.

Sleep quality was adequate across all groups, with 100% reporting at least 7 h of sleep per night. However, sleep restlessness was reported by 16% of the never-exposed, 56% of the transiently exposed, and 78% of the continuously exposed children.

Family factors indicate that most families have a middle socio-economic status: 98% of never-exposed families, 100% in the transient exposure group, and 97% in the continuous exposure group. Regarding maternal education, 80% of mothers in the never-exposed group held university degrees, compared to 40% in the transient and 77% in the continuous exposure groups. Although there were numerous professional mothers in the study, most of the families belonged to a middle socio-economic status. This situation is explained by the location of the study in the municipality of Cárdenas, which is 10 km from Varadero, in a tourist area. Most mothers from Cárdenas work in Varadero, and many of them are professionals. However, most of them do not work as professionals in the tourism sector. They work in other jobs such as laundry, cooking, bartending, cleaning, security, and administration. As a result, many working mothers in the tourism industry earn non-professional salaries, even if they have university degrees.

Lastly, children’s behavior varied: 80% of children who had never been exposed to smoke participated in adult-directed play, compared with 68% and 79% of children who had been transiently and continuously exposed, respectively. Daytime restlessness was observed in 9% of children who had never been exposed, 52% of those who had been transiently exposed, and 76% of children who had been continuously exposed.

Table 2 documents the relationship between household smoke exposure and brain health indicators. The Durbin–Watson test (2.19) indicates that there is no autocorrelation in the residuals. Collinearity is acceptable, with tolerances greater than 0.1 and low VIFs. In addition, the skewness (0.44) and kurtosis (2.59) values suggest that the residuals have an asymmetric distribution close to 0, with no normality problems. The ANOVA analysis found significant values (F = 3.43; *p* = 0.003). In the analysis of the linear regression coefficients, the only significant variable was sleeping quality (−0.13, *p* = 0.02). This result suggests that as exposure to household smoke increases, sleep quality problems increase.

Analysis using the *t*-test between the groups of children temporarily and continuously exposed to household smoke revealed significant differences in several areas: working memory (*p* = 0.009; Cohen’s d = 0.62), inhibition (*p* = 0.001; Cohen’s d = 0.81), cognitive flexibility (*p* = 0.001; Cohen’s d = 0.76), and oral language development (*p* = 0.018; Cohen’s d = 0.68), with effect sizes ranging from medium to large. In contrast, no significant differences were found for fluid intelligence (*p* = 0.58; Cohen’s d = 0.13), brain health (*p* = 0.390; Cohen’s d = 0.20), or sleep quality (*p* = 0.118; Cohen’s d = 0.37), which had small effect sizes.

Table 3 reports on results that analyze how family and its components influence the relationship between brain health and exposure to household smoke, both transient and continuous, in the home. The Durbin–Watson test result of 1.20 suggests that there is no autocorrelation present in the residuals. Collinearity is deemed acceptable, as indicated by tolerances exceeding 0.1 and low VIF values. The skewness (0.21) and kurtosis (2.03) metrics imply that the residuals exhibit a distribution that is slightly skewed but close to normal, with no significant normality issues. The ANOVA results are significant (F = 5.31; *p* = 0.001).

The analysis reveals a negative correlation between both transient and continuous exposure to household smoke from maternal sources in the home (SMOKEi) and brain health (BRAINiAge). Specifically, increased exposure is linked to a decline in brain health (b = −0.13, SE = 0.03, 95% CI [−0.07; −0.19], and *p* < 0.001). Conversely, a positive relationship with family status (FAMILYi) indicates that improved family conditions are associated with better brain health outcomes (b = 1.51, SE = 0.65, 95% CI [0.20; 2.81], and *p* < 0.05).

Individual characteristics of children (CHILDi) also had a significant positive correlation with brain health (b = 0.46, SE = 0.03, 95% CI [0.40; 0.52], and *p* < 0.001). Furthermore, family socioe-conomic status is strongly positively associated with brain health, suggesting that higher socio-economic status correlates with improved brain health (b = 0.84, SE = 0.04, 95% CI [0.74; 0.93], and *p* < 0.001). Maternal education contributes positively as well (b = 0.08, SE = 0.04, 95% CI [0.00; 0.15], and *p* < 0.05).

In contrast, no significant associations were found between variables such as the economy (ECONOMYi), playing with other children, discipline, daytime restlessness, or the number of family members.

Furthermore, transient and continuous exposure to household smoke has a more negative effect on brain health in boys (b = −0.19, SE = 0.04, 95% CI [−0.11; −0.28], and *p* < 0.001) compared to girls (b = −0.01, SE = 0.03, 95% CI [−0.05; −0.08], and *p* > 0.20). Similarly, daytime restlessness was significantly different between boys (b = −0.12, SE = 0.05, 95% CI [−0.01; −0.22], and *p* < 0.05) and girls (b = −0.04, SE = 0.08, 95% CI [−0.14; −0.22], and *p* > 0.34), indicating that household smoke exposure leads to a higher level of restlessness in boys than in girls.

## 4. Discussion

Cuba’s economic system presents unique challenges. Although classified as a middle-income country, compared with other socialist economic model states that underwent similar timely revolutions, its economic indicators of productivity have been consistently in deficit as that of a lower-income country since 1960 [50]. Meso-Lago [50] summarizes the outcomes: (a) between 2009–2020, the annual GDP grew 7.5% in China, 5.8% in Vietnam, and only 0.96% in Cuba. (b) The 2019 gross national product was USD 10,140 in China, USD 2741 in Vietnam, and USD 384 in Cuba. (c) Cuba’s economy showed comparative deficits by 10.9% instead of increments. Thus, children are born in a country with significant challenges in its distribution of wealth, affecting employment opportunities and consequential inequalities in health and dental care [51]. More than ever, there is a solid case for optimizing cerebral health in the next generation of its children, who need favorable, if not prime, conditions for health and achievement opportunities. Household indoor air environments are a good start.

While prior research has primarily focused on the risks of maternal smoking during pregnancy and household smoke in non-LMICs [6,7], it remains crucial to identify associated developmental risks in socio-demographic and socio-political environments that are less clear. This cross-sectional study aimed to investigate the risks associated with prolonged exposure to secondhand smoke in the home, produced by mothers, on cognitive and non-cognitive outcomes that indicate neurodevelopmental health. More specifically, we examined executive function, language development, fluid intelligence, and sleep quality during the first six years of life. Maternal smoking, viewed as a proximal factor in children’s micro-environment, negatively influences children’s health and learning. Additionally, broader economic conditions (distal factors) seem to worsen these risks [52].

In the case of Cuba, factors such as maternal smoking, education, and family socio-economic status greatly affect the cognitive and non-cognitive brain health of children exposed to tobacco smoke at home. However, the overall economic context, as indicated by GDP per capita and Human Development Index data, did not significantly affect children’s brain health, probably due to the low variability between the families analyzed since 97% of them had a medium Cuban income and the rest belonged to the high-income category. In future research, it would be advisable to consider this aspect and seek a balance between the number of low-, medium-, and high-income families. It is essential to conduct this analysis in the future, as economic indicators in Cuba have not performed adequately over the past 10 years [53]. The family environment reflects these economic weaknesses, which impact the child’s development, even though the family is motivated to promote their growth.

Our observations suggest that secondhand smoke exposure at home affects both cognitive factors of the brain health of children (executive function, language development, and fluid intelligence) and non-cognitive factors (sleep quality). Furthermore, while the amount of tobacco exposure is similar for both boys and girls in terms of hours and days per week, the negative effects are more evident in boys, as suggested by their higher levels of restlessness compared to girls.

Our findings indicate that most children exposed to household smoke at home manifest restlessness, both during the day and at night, which is consistent with other studies that have found impaired sleep quality in children exposed to household smoke [19,20]. However, the family environment in Cuba could play a protective role, as it was observed that most parents had a high level of education, 97% of families belonged to a middle socio-economic class, and 90% of families had more than four members, which could favor the child’s education due to the support of other members.

Despite family conditions, the duration and frequency of exposure to household smoke best explain the relationship between brain health and family environment, regardless of income level. Most of the families in the study were middle-income, suggesting they have resources to cope with family problems. However, many of these families expose their children to household smoke on a continuous basis (3 to 7 days per week and more than 3 h per day), suggesting that while household income may offer some protection, continued exposure has the potential to exacerbate brain health problems and behavioral problems in children. Thus, protective and risk factors that influence child development coexist in these families, and the duration and frequency of exposure are important determinants of a child’s brain health.

Previous studies in Latin America have also documented similar findings regarding smoking parents [27,28]. Both our research and that of Bernabe-Ortiz [27] indicate that a significant number of children are subjected to secondhand tobacco smoke in their homes. Bernabe-Ortiz [27], using data from the Global School-based Student Health (GSHS) surveys conducted between 2010 and 2018, indicated that 60% of children in 18 countries in the region are exposed to this smoke. Additionally, both our study and that of Prado-Galbarro [28] note that anti-smoking campaigns have effectively reduced smoking rates among adults and adolescents in Cuba and the broader region. However, it is essential to continue advancing in the eradication of this habit from a development science perspective, both in Cuba and throughout Latin America.

Our findings highlight three important contributions to developmental science for developing countries regarding secondhand smoke and brain health in preschool children. First, it confirms that children exposed to secondhand smoke in early childhood are a vulnerable population, not only for health science but also for developmental science. Observations from the study suggest that children’s cognitive function and sleep quality are impaired during the first 6 years of life when the mother smokes, which is a negative indicator for the child’s school readiness and mental health. In this sense, maternal smoking is a risk factor for the development of self-regulation, working memory, and sleep quality in children.

Second, the duration and frequency of exposure to household smoke negatively affect the family’s protective role in child development. In families of middle socio-economic status, problems in both cognitive and non-cognitive functions are observed in children exposed either continuously or transiently. This suggests that health initiatives and anti-smoking campaigns should target all Cuban households with smoking parents since children between the ages of 4 and 6 are passive smokers, do not complain, and do not understand what they are inhaling, even when they are near their mothers.

Thirdly, Duncan’s [54] equation provides a suitable theoretical and methodological framework for investigating brain health in early childhood in low- and middle-income countries from a developmental sciences perspective. This is particularly relevant when analyzing the impact of household smoke in the home and its influence on the child’s bio-ecological development. It is essential to consider brain health from cognitive factors, such as executive function, oral language development, and fluid intelligence, as well as from non-cognitive aspects, like sleep. It is also important to account for confounding factors, such as the family’s socio-economic status, the family environment, and teacher–parent relationships, among others.

## 5. Conclusions

Although every child born faces economic challenges that will require enormous courage and economic reform, the Cuban state has shown a strong political will to ensure optimal child development and well-being [55]. This is especially the case for education and coverage and mother–child health care. However, once born, children need a proper environment to achieve proper development and well-being. The results of this study conclude that mothers who smoke are more likely to put their 5- to 6-year-old children’s brain health at risk compared to non-smoking mothers. Continuous exposure to tobacco smoke (5 to 7 days a week for 2 to 3 h) and transient exposure (two to three times a week for 2 to 3 h) can negatively affect children’s sleep quality, causing restlessness during sleep, movements that awaken them, and multiple sleep interruptions. Furthermore, cognitive problems, such as executive and language functions, can result not only from poor sleep quality but also from daily exposure to secondhand smoke.

Furthermore, the socio-economic conditions of Cuban families are significantly linked to children’s brain health, as expected. However, many smoking mothers were found to have high wages and professional occupations, suggesting that other factors should be explored in future research, especially in relation to family stress and the father’s smoking habits. This aspect is relevant because Cuban families have adequate educational resources, which makes it surprising that many families with high levels of education expose their children to secondhand smoke. Informing parents and caregivers that tobacco smoke is harmful to children’s development represents an important preventive intervention strategy that is both actionable and modifiable.

## 6. Limitations in the Study

The current study acknowledges several limitations relevant to the Cuban context that warrant consideration for future research. First, we assessed maternal smoking as though fathers do not exist, neither in the home nor in the family. Cuba has a robust early childhood education system that encourages children to attend either a childcare center or participate in home-based programs [56]. Consequently, most children aged 0–5 in Cuba do not stay home with their mothers. This system is designed to provide quality care and education for all children, regardless of family income. This means that our estimates underestimate the risk, are thus, at the very least conservative, and based on one parent and exposure on weekday afternoons and evenings and weekends.

Second, although the interviews conducted in our study offered valuable insights into family circumstances, they were inadequate for fully assessing maternal smoking habits and children’s sleep quality, potentially limiting the generalizability of our findings. Previous research has utilized questionnaires to evaluate infant sleep quality [25], detect home nicotine exposure [14], and analyze maternal anxiety in relation to smoking [55]. These approaches emphasize the importance of incorporating diverse qualitative measures in future studies.

Third, linear regression identified associations between key variables (brain health indicators and secondhand smoke exposure), although causal relationships between them could not be established. This indicates that the results show correlations but do not imply causality. In the future, a causal analysis between secondhand smoke in the homes of Cuban families and brain health indicators is needed, which could provide the health system and the educational sector in Cuba with more information to intervene in families with smoking parents.

This study suggests that the most vulnerable children in low- and middle-income countries are likely more vulnerable than what is reported in this study. Given Cuba’s ongoing weak economic, educational, and health challenges on the ground, it would be imperative to consider brain health through various lenses, including executive function, language development, sleep quality, parenting styles, and family stress. Ultimately, our findings serve as a catalyst for advancing community development with parent information campaigns in Cuba, focusing on long-term outcomes that foster reflection on effective practices and inform strategic decision-making, thereby contributing to the improvement of educational and developmental outcomes of Cuban children. Promotion and protection of these factors will promise better access and control over wealth, even in a deprived country like Cuba.

## Figures and Tables

**Table 1:** Sample characteristics according to household smoke exposure.

Days in the Week of Exposure to Household Smoke at Home			
	Never Exposed (no Day a Week) (n = 535)	Transient Exposure (2–3 Days) (n = 25)	Continuous Exposure (5–7 Days) (n = 67)
Age (months) (mean score)	72 (8.36)	82.50 (4.57)	80.82 (4.96)
Sex (% boys)	323 (58%)	17 (68%)	24 (36%)
Index of household exposed risk (% high (≥1) and low (<1)).	0 (100%)	High: 8 (32%)	High: 67 (100%)
Maternal smoking (% smokers)	0 (100%)	25 (100%)	67 (100%)
Hours per day of exposure to household smoke (% high (≥4 h), medium (2 to 3 h), and low (≤1 h).	0 (100%)	High: 2 (8%) Medium: 6 (24%)	High: 7 (10%) Medium: 17 (25%)
*Brain Health Indicators (mean score)*	4.34 (2.55)	4.80 (1.63)	5.78 (2.39)
Working memory (mean score)	12.87 (2.62)	13.43 (1.97)	14.89 (2.21)
Inhibition (mean score)	11.53 (2.32)	12.04 (1.51)	13.71 (1.74)
Cognitive flexibility (mean score)	10.74 (2.17)	11.20 (1.44)	12.69 (1.69)
Oral language development (mean score)	43.69 (8.19)	46.63 (5.22)	50.36 (4.21)
Fluid intelligence (mean score)	15.62 (1.66)	16.50 (1.75)	16.85 (1.48)
Sleep quality			
Child’s sleep hours (% ≥7 h during the night)	535 (100%)	25 (100%)	67 (100%)
Sleep restlessness (% present)	87 (16%)	14 (56%)	52 (78%)
*Family Factors*			
Family socio-economic status (% medium)	522 (98%)	25 (100%)	65 (97%)
Number of family members (% ≥four members).	489 (91%)	25 (100%)	61 (90%)
Maternal education (% University)	428 (80%)	10 (40%)	51 (77%)
*Child Factors*			
Playing with other children (% adult-directed games)	427 (80%)	17 (68%)	53 (79%)
Discipline (% bad discipline)	51 (9%)	2 (8%)	17 (25%)
Daytime restlessness (% present)	50 (9%)	13 (52%)	51 (76%)

**Table 2:** Unstandardized regression coefficients (with standard errors) indicating the relationship between household smoke exposure (hours per day of exposure) and brain health, including its components.

*Independent Variable*	*SMOKEi* *b (SE)* *[95% CI]*	*Independence Sample t-Test*		
		*t*	*p*	*Cohen’s d*
*Brain Health Indicators*	−0.11 (0.06) [−0.25; −0.02]	−0.86	0.390	0.20
Working Memory	−0.14 (0.23) [−0.60; −0.31]	−2.65	0.009	0.62
Inhibition	−0.41 (0.62) [−0.82; −1.66]	−3.49	0.001	0.81
Cognitive Flexibility	0.03 (0.71) [1.38; 1.44]	−3.28	0.001	0.76
Oral Language Development	−0.08 (0.13) [−0.35; −0.19]	−2.93	0.018	0.68 ^a^
Fluid Intelligence	−0.79 (0.06) [−0.68; −0.09]	−0.58	0.58	0.13
Sleep Quality	−0.13 * (0.05) [−0.01;−0.25]	−1.57	0.118	0.37

Note: b = beta coefficient, CI = confidence interval, and SE = standard error. * *p* < 0.05 In the linear regression analysis, data from 92 children with continuous and transient exposure were used, while 535 children who were never exposed were excluded using SPSS’s “variable selection” option. Independence Sample *t*-test between group A (children with continuous exposure to household smoke during the week) and group B (children with transient exposure to household smoke during the week). ^a^ The oral language development variable does not comply with the homogeneity of variances. The BRAINi variable represents the average of four key variables (executive functions, linguistic functions, fluid intelligence, and sleep quality). These scores were converted into Z scores.

**Table 3:** Unstandardized regression coefficients (with standard errors) reflecting the relationship between confound controls and brain health indicator outcomes.

*Independent Variable*	*BRAINiAge* *b (SE)* *[95% CI]* *(n = 92)*	*Boys BRAINiAge* *b (SE)* *[95% CI]* *(n = 51)*	*Girls BRAINiAge* *b (SE)* *[95% CI]* *(n = 41)*
Smoke_i_	−0.13 *** (0.03) [−0.07; −0.19]	−0.19 *** (0.04) [−0.11; −0.28]	−0.01 (0.03) [−0.05; −0.08]
Economy_i_	−0.71 (0.65) [−2.02; −0.59]	−0.39 (0.81) [−1.23; −2.03]	−0.78 (0.78) [−2.36; −0.80]
Family_i_	1.51 * (0.65) [0.20; 2.81]	0.38 (0.80) [1.24; 2.00]	1.56 * (0.78) [0.04; 3.16]
Child_i_	0.46 *** (0.03) [0.40; 0.52]	0.43 *** (0.40) [0.35; 0.51]	0.36 *** (0.03) [0.28; 0.44]
Playing with other children	0.05 (0.08) [0.02; 0.19]	0.07 (0.07) [0.21; 0.07]	0.04 (0.08) [0.12; 0.20]
Discipline	−0.01 (0.03) [−0.05; −0.08]	−0.02 (0.03) [−0.09; −0.05]	−0.01 (0.04) [−0.10; −0.08]
Daytime restlessness	0.006 (0.04) [0.09; 0.10]	0.12 * (0.05) [0.01; 0.22]	0.04 (0.08) [0.14; 0.22]
Family Socio-economic status	0.84 *** (0.04) [0.74; 0.93]	0.77 *** (0.05) [0.65; 0.88]	0.80 *** (0.06) [0.66; 0.94]
Maternal education	0.08 * (0.04) [0.00; 0.15]	0.02 (0.05) [0.09; 0.14]	0.04 (0.05) [0.03; 0.13]
Number of family members	0.04 (0.04) [0.05; 0.13]	0.04 (0.05) [0.07; 0.16]	0.03 (0.05) [0.08; 0.14]

Note: b = beta coefficient, CI = confidence interval, and SE = standard error. BRAINiAge = a1 + ß1 SMOKEi + γ1 ECONOMYi + γ2 FAMILYi + CHILDi + eit. * *p* < 0.05, and *** *p* < 0.001. In the linear regression analysis, data from 92 children with continuous and transient exposure were used, while 535 children who were never exposed were excluded using SPSS’s “variable selection” option.

## Data Availability

The data presented in this study are available on request from the corresponding authors.
